# Navigating the Unseen City: Town Planners, Architects, Ophthalmic Professionals, and Charity Opinions on Navigating of the Built Environment with a Visual Impairment

**DOI:** 10.3390/ijerph19127299

**Published:** 2022-06-14

**Authors:** Laura N. Cushley, Neil Galway, Katie Curran, Tunde Peto

**Affiliations:** 1 Centre for Public Health, Queen’s University Belfast, Grosvenor Road, Belfast BT12 6BA, UK; k.curran@qub.ac.uk (K.C.); t.peto@qub.ac.uk (T.P.); 2 School of the Natural and Built Environment, Queen’s University Belfast, Stranmillis Road, Belfast BT9 5AG, UK; n.galway@qub.ac.uk

**Keywords:** visual impairment, sight loss, navigation, towns and cities, built environment, streetscape

## Abstract

People with a visual impairment often find navigating around towns and cities difficult. Streetscape features such as bollards, street cafés, and parked cars on pavements are some of the most common issues. in this paper semi-structured interviews were conducted with stakeholders including built environment professionals, visually impaired individuals, ophthalmic professionals, and sight loss charities. All stakeholders felt there were barriers and enablers to navigating streets with a visual impairment. Stakeholders agreed these can have an impact on the daily lives of those with a visual impairment. While built environment professionals knew of policies and guidance around accessibility for people with a visual impairment, there was a lack of professional knowledge about the spectrum of visual impairment. Despite this, stakeholders felt these small changes could have a positive impact, making accessible cities for all. A collaborative approach to streetscape design and further education could help create better environments for all.

## 1. Introduction

Towns and cities are the settings for our everyday lives. The ability to live and navigate these spaces allows us to have social interaction, sustain relationships, and access necessities and our workplaces [1,2]. There are multiple stakeholders in urban landscapes, all of which have different wants and needs. Our urban landscapes affect health and wellbeing [3,4], especially for people with a disability, namely a visual impairment. Traditionally, vision is recognised as the most important sense when experiencing urban environments [5,6,7,8]. Therefore, when a person’s vision is impaired, they often find it difficult to navigate towns and cities safely and effectively. Moving and navigating around towns and cities is often described as a nerve-wrecking experience [9] fraught with dangers, both actual and imagined [10]. Without independent mobility, visually impaired people can often be affected physically and emotionally, which in turn affects their quality of life.

The current literature shows there are several known problems that make the built environment “hostile” [11]. These include street furniture, dropped kerbs, street cafes, and advertisement boards (A-boards). In addition, other “non-permanent” problems include parked cars on the pavement, wheelie bins, tree roots, and uneven/broken surfaces. Shared space is also a very contested issue with visually impaired users as it relies on eye contact as the main social interaction to maintain safe navigation [12]. 

These potential problems within our streetscape restrict mobility and movement for the visually impaired members of our society. Visual impairment is an established risk factor for independence [13] and is one of the most significant causes of loss of independence among older people [14]. A 2014 project called “Cities Unlocked” suggested 180,000 people with visual loss rarely leave their houses [9] and most do not like to travel alone [15]. Going out into the built environment creates feelings of anxiety and vulnerability [9]. This can all lead to social isolation or exclusion, which in turn can cause mental, emotional, and physical issues, especially in older adults [16,17]. 

To prevent this emotional and physical burden, built environment professionals are tasked with making areas that are attractive and usable for all. Design approaches must take into account the “diversity of human abilities and conditions” [18], as well as differing opinions, needs, and wants. 

Some of the literature suggests a collaborative approach to the design of the built environment is good practice [19,20]. Collaboration in the creation of built environments is a concept that has been around for many years. Godschalk and Mills introduced and advocated the concept of a collaborative approach to planning spaces in 1966, stating “meaningful and effective planning must be based on a two-way communication flow between the public and planning agency” (Page 88). This was further supported by Arnstein [21], who argued for more substantial input by the general public into planning. While both society and planning have changed markedly since then, “planning as a collaborative process” remains a core principle of the profession.

While the planning profession recognises the concept of collaborative planning with the general public, it is also important that built environment professionals, including planners and architects, work together to create accessible and attractive environments for all. Architects are also regarded as key agents in the design and creation of built environments; this is why it is important for both users of the environment and professionals to share concepts and collaborate [22]. 

Despite this collaborative design approach becoming increasing discussed and accepted, there is a paucity of literature on stakeholder opinions from built environment professionals, ophthalmic professionals, charities, and members of the visually impaired community. This paper aims to establish key stakeholder, including town planners and ophthalmic professionals, opinions on navigating the built environment with a visual impairment, as well as potential future solutions. 

## 2. Materials and Methods

### 2.1. Ethical Permission 

This study was reviewed and approved by the Medicine Health and Life Sciences Research Ethics Committee, Queen’s University Belfast (approval number MHLS 20_67).

### 2.2. Study Design

This study forms part of the larger ‘NaviSight study’, which encompasses stakeholder views on navigating the built environment with a participant-based study. This part of the study focuses on interviews with professionals and the visually impaired community, gauging their views on navigating towns and cities with a visual impairment. 

Prospective interviewees were contacted via email through personal contacts, clinical department leads, department of communities, and infrastructure and city councils. This study was based on the island of Ireland, and potential stakeholders were contacted across Northern Ireland and the Republic of Ireland. A majority of respondents worked in or close to Belfast and Dublin and were interviewed between December 2020 and March 2021.

Semi-structured interviews were conducted with 20 stakeholders, including built environment professionals, visually impaired individuals, ophthalmic professionals, and sight loss charities. Below is a list of interviewees:

- Visually impaired people from Northern Ireland (NI), *n* = 3
- Town planners from NI, *n* = 3
- Architects from NI, *n* = 4
- Ophthalmologists from NI, *n* = 2
- Ophthalmologists from the Republic of Ireland (ROI), *n* = 2
- Optometrists from NI, *n* = 2
- Sight loss charities in NI, *n* = 3
- Sight loss charities in ROI, *n* = 1

Semi-structured interview guides were used with questions focused on the interviewee’s knowledge and awareness about visual impairment, shared spaces, transport, and existing policy/guidance. Interviews began with questions surrounding potential issues in the built environment for people with a visual impairment. Interviewees were then asked about issues such as shared space, pedestrian crossings, public transport, and streetscape lighting. 

Interviewees were also asked about their public awareness of visual impairment. Planners and architects were asked about awareness in their profession. Interviewees were asked whether they would like to see more education on visual impairment and how this could be achieved. 

Ophthalmic professionals and charities were asked if patients with a visual impairment have ever talked about problems moving around towns and cities. In addition, planning professionals and architects were asked about their awareness of any policy and guidance that relates specifically to visual impairment. 

Interviewees were encouraged to talk freely about what is important to them and questions were asked only to guide the interview. Data saturation was met with 20 interviews, given the depth of similar data given by many stakeholders. We defined data saturation as consecutive interviews no longer adding additional data. 

### 2.3. Demographic Information 

Planning professionals ranged from having 3 years of experience to having over 20 years of experience in the field. Planning professionals were also from several different departments, including transport policy, department of communities, and department of infrastructure. Ophthalmic professionals included clinical and research ophthalmologists and optometrists with 10–40 years of experience. Architects were from the public or private sectors with 10–45 years of experience in the field. Visually impaired individuals included people with loss of central and peripheral vision and loss of vision in one eye.

### 2.4. Data Analysis

**Figure 1:**
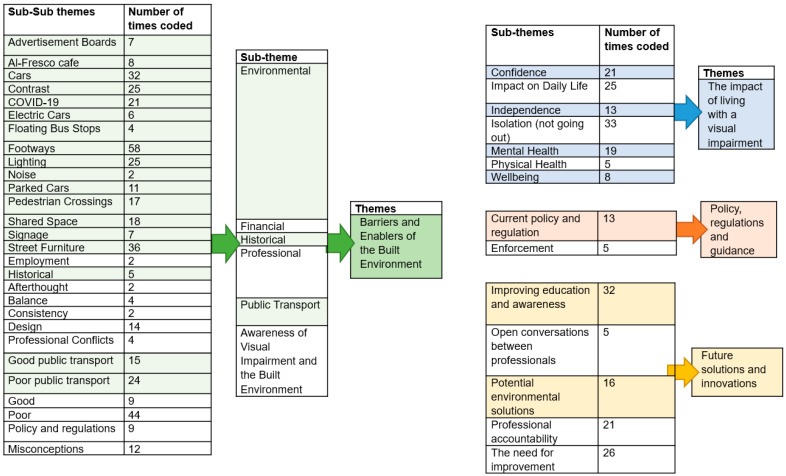
Main themes, sub-themes and sub-sub themes from data analysis.

All interviews were transcribed and imported into NVivo software 1.6.1, QSR International (Burlington, VT, USA) for analysis by a PhD student (LC). NVivo is a computer software package for qualitative analysis that helps organise and analyse qualitative research including interview transcripts. On analysis, each transcript was assigned a case classification—built environment professionals (architects and planners), ophthalmic professionals (optometrists and ophthalmologists), charities and visually impaired individuals. Transcripts were coded following Braun and Clarkes’ thematic analysis techniques, and these codes were used to create themes, subthemes and sub-sub themes according to the most prominently discussed issues. The frequency of codes is presented in Figure 1 to illustrate the most common themes and subthemes within the data. 

Twenty-five percent of the transcripts were then analysed in NVivo software by a secondary coder, a research fellow (K.C.). The process of analysis was identical to that of the primary analyst. One transcript was randomly selected from each case classification to ensure all viewpoints were represented. Upon completion of the coding analysis in NVivo, a thematic analysis table was completed for comparison. 

Both analysts met to establish agreed themes and subthemes, and a senior researcher (TP) adjudicated and agreed upon these final themes and subthemes.

For analysis, architects were given the code ARC, planners were given the code PLA, visually impaired individuals were given the code VIP, charities the code CHA, and ophthalmic professionals the code OPH. 

## 3. Results

Four main themes were identified, namely: (1) barriers and enablers of the built environment; (2) policy, regulation, and guidance; (3) the impact of living with visual impairment; and (4) future solutions and innovations. Twenty-one subthemes were also identified with further sub-subthemes (Figure 1). Where the number of times something was discussed is in the manuscript, counts were taken across all transcripts; therefore, if a person discussed it more than once, this was counted as a new mention. 

### 3.1. Theme 1: Barriers and Enablers in the Built Environment

#### 3.1.1. Environmental 

Interviewees talked about environmental barriers such as advertisement boards, al-fresco dining, colour contrast, COVID-19, electric cars, footways, lighting, and shared space. 

A-boards were negatively discussed by charities and visually impaired people who talked about how they create narrow pavements and street clutter, which can be dangerous.

“A lot of our town centres [have] narrow streets, [streets] are cluttered in terms of advertising boards out the front”.

(CHA03)

‘‘I think it’s really dangerous that people put [advertisement boards] out for their shops”.

(VIP01)

An ophthalmic professional also stated A-boards are unpredictable and ‘unpermenent’ in nature, which make them particularly hazardous.

“Sight[/advertisement] boards outside shops that aren’t always there, you know people stick them out and then they take them in”.

(OPH04)

COVID-19 saw alfresco dining becoming more prominent with many temporary licenses issued by councils. While charities and other stakeholders understood this was necessary due to the economy opening up again, there are associated dangers.

“Street furniture in terms of cafés going right out into the street which is understandable as they are trying to get as many tables in as possible”.

(CHA03)

‘‘[we] aren’t opposed to café culture it just needs to be done in the right way where it isn’t impacting upon people, [especially] as we move through in a couple of months and cafes start opening again”.

(CHA03)

These dangers include problems with definition and cordoning street cafés to allow for easy manoeuvrability for people with visual aids.

“If someone is using a mobility cane… on the ground …cordoned off area[s] at some cafes [are difficult], the barrier doesn’t [go to ground level]… they might miss it”.

(CHA03)

There were also concerns that with more COVID-19 restrictions lifting, “it’s only going to get worse” (OPH02).

All visually impaired people, charities, architects, and town planners felt cars and the transport hierarchy negatively impacted the safety and navigability of the built environment for the visually impaired. Cars were mentioned 28 times across the interviews, all negatively. 

Stakeholders felt the transport hierarchy was not followed in NI, with it being “all about the motorist” (VIP01).

‘‘There is a transport hierarchy supposedly in place [where pedestrians and disabled people [should come] first and the private car last but in my experience, it is the private car first and foremost”.

(PLA02)

Others reflected that by giving cars so much space, everyone else including cyclists have to use pavements.

“The idea that we give so much space to cars and everyone else has to jostle on the pavement in fear of their death”.

(ARC01)

“How much of the city is given to cars and if you step off the pavement you may as well be falling 1000 feet”.

(ARC01)

The speed of cars was also deemed an issue, frightening people with a visual impairment.

“Cars are driving so so fast. It’s pretty hair raising. I just want them to slow down”.

(VIP01)

Colour contrast was also widely discussed by all stakeholders (mentioned 25 times), who agreed there should be more appropriate contrast on streets and in buildings to allow for easier navigation.

“Yeah, the big issue is the contrast in environments, particularly in built up environments, you have the different shades of grey rather than the high contrast things or tactile things”.

(OPH05)

The grey hue of built environments was deemed problematic.

“I find it hard to find edges of pavements and the road. I think all those little things like the colour of the pavement and the colour of the road”.

(VIP01)

There are also several examples of using colour contrast in inappropriate scenarios.

“Take one example—the aesthetic of the building in terms of interiors—you may want to have the same colour on your skirting boards as well as your door and door surround [but] that means that people cannot define where the door is”.

(ARC01)

“Architects often use things like ‘snazzy floors’ and contrasting colours on a flat surface (which can look like level changes and holes in the ground to someone with a visual impairment)—[they] use them in the wrong place”.

(PLA01)

Footways were mentioned 58 times, with the main barriers being level changes, narrowing of footways, kerb heights, enforcement, and maintenance after schemes/roadworks.

“This narrowness of streets also make them crowded and dangerous”.

(PLA02)

“Things like public realm tactile paving not being finished.’ Rubbish and overgrown shrubbery on the street….lack of tactile paving….tactile paving wasn’t finished or it had been damaged and not repaired [and] ‘maintenance management”.

(CHA03)

“Dipped or cracked pavement”.

(OPH02)

“Inappropriately maintained footpaths”.

(OPH06)

#### 3.1.2. Another Barrier Was Soiling on Footways

“Soiling—explosion in the number of dogs who have been bought and walked in the pandemic and the soiling has become a real issue down here—I mean I don’t use a cane and it’s difficult”.

(OPH02)

“Dog fouling—[affects people with a] visual impairment [more]”.

(VIP03)

Lighting was discussed by half of interviewees; 80% had negative comments suggesting lighting was poor or lacking in areas, while others mentioned current schemes to improve street lighting.

“Yes, there’s plenty of street lighting however places like the comber greenway are not illuminated”.

(PLA02)

“There are debates around street lighting—there is too much lighting in some areas which creates light pollution but there are also areas which don’t have enough lighting”.

(PLA03)

“There could be better street lighting in a lot of Belfast”.

(VIP03)

“We really need more [light]”.

(VIP01)

Lighting is not only beneficial for people with a visual impairment, it is good for everyone, especially for safety.

“Light is good for everyone, it makes a place feel safe and welcoming”.

(PLA01)

“At least I can see lights and shadows when there are streetlights so it helps”.

(VIP02)

Stakeholders felt there were positives and negatives to shared space. Visually impaired persons and charities had mostly negative thoughts about it, while planners and architects were in favour. Architects and planners saw it as a way to slow down cars and take back ownership from a car-centric town or city.

“I do like shared space because I see it almost as people taking back space—accidentally been given to cars”.

(ARC01)

“Shared spaces are good as getting rid of cars in large parts of town and city centres would be a good thing”.

(PLA01)

Planners and architects argue the problem is not the concept of shared space itself, but the design of the scheme and the behaviour of people.

“Shared space is a good thing although I recognise it does have its challenges however, this is not down to the concept of shared space itself”.

(PLA02)

“Shared space is definitely a good thing, the issues arise when the shared space is badly designed”.

(PLA03)

Visually impaired people and charities felt shared space was often “a big no no” (CHA01), with cars thinking they have priority in these areas.

“Planning of Carrickfergus—pedestrians are allowed to walk in the town and the car drives fast down the pedestrian streets and we just have to get out of the way immediately because they are coming through—they think they have priority”.

(VIP01)

While stakeholders felt there were enough pedestrian crossings in some areas, one of the biggest problems was many pedestrian crossings’ audible signals were either broken or had been turned off.

“For some reason [they] turn off the audio signal at traffic lights because … its noisy and keeps [people] up at night,…. [they are] not thinking of the impact of that crossing for people with sight loss”.

(CHA02)

“Beeping crossings—my own experience would be very often they actually don’t function”.

(VIP01)

If no accessible signals are working on a crossing, or there is no accessible crossing, it makes it extremely difficult to cross the road safely.

“Sometimes the green man doesn’t work and I can’t hear it, the [tactile cone] signal on the bottom doesn’t turn”.

(VIP01)

One of the most prominent issues discussed was street furniture, which was discussed by 11 interviewees and mentioned 36 times.

“Street furniture is historically one of the big ones and certainly one that we would get a lot of complaints or concerns about”.

(CHA03)

Bollards, bikes, random posts, gates, and bins in streets were deemed as some of the biggest obstacles.

“Bollards—I walk into bollards—single most painful thing in my life—the agony…”.

(VIP01)

‘‘My worst nightmare is bollards and they are my biggest enemy’’.

(VIP03)

Parked cars on pavements were discussed as being a problem for people with a visual impairment and for people in wheelchairs or pushing prams.

“Sometimes I’m trying to push my mum in her wheelchair and all the cars are parked on the pavement, it’s just difficult’’.

(VIP01)

Floating bus stops were also discussed by planners and charities as a very current issue.

“Bus lanes—floating bus lanes—bus borders, all these types of things [are problems]”.

(CHA03)

Electric cars and their minimal noise emission were also deemed to be a problem.

“Electric cars are a scary nightmare—I don’t hear a thing”.

(VIP01)

Alternatively, planners felt that reduced noise and air pollution were overall positive,

“So its balancing the benefits, etc., of everything”.

(PLA03)

#### 3.1.3. Historical 

Stakeholders agreed that environmental problems often build up over time. Historically, town and city planning did not take into account people with disabilities.

“Historically the planning of our towns and villages didn’t take into account people with disabilities, it’s only recently within the last couple of decades that you know we’re at least starting to get to grips with it—that’s kind of my sense of it”.

(CHA03)

The “old towns with old streets and pavements” (OPH02) often historically have a lot of these environmental barriers. 

#### 3.1.4. Financial 

There are also financial implications that stem from visual impairment and the inability to move around towns and cities independently and confidently.

“Only 16% of people at working age with sight impairment are employed and one of the biggest barriers to employment is moving around, getting to and getting from where you work”.

(CHA02)

#### 3.1.5. Professional 

Some stakeholders discussed the barrier of professional conflicts, for example, one department thinking another department will deal with that issue.

“Planning officers often leave the problem to consultations and other departments to deal with”.

(PLA03)

In addition, some felt some professional worlds have a “toxic” attitude (PLA01), with academics and lecturers teaching you to follow the regulations and not to think outside the box.

“A lot of lecturers want you to follow the regulations not conceptual architecture”.

(PLA01)

Stakeholders agreed that some built environment professionals create environments for “normal people”.

“I do however think that architects have an emphasis on what you would say are ‘normal people”.

(ARC02)

All built environment professionals agreed there needs to be a people-centric design in towns and cities, with disabilities and other factors considered in the early stages.

“Design for [user-environments] should be being designed with visually impaired in mind”.

(PLA01)

“People-centric design approach is good and helps all people with disabilities (including visually impaired)”.

(PLA01)

There does however need to be a balance of people’s needs and that can be difficult to achieve.

“The key word in planning is balance…It’s difficult to strike the balance”.

(PLA03)

#### 3.1.6. Awareness of Visual Impairment and the Built Environment 

Overwhelmingly, 19 out of 20 people stated there was not enough awareness about visual impairment and the built environment. Issues with awareness of the varied level of sight loss and misconceptions was seen by charities, built environment professionals, ophthalmic professionals, and visually impaired people.

“A lot of professionals aren’t aware for example that only 5% of visually impaired people are actually completely blind”.

(PLA01)

“It’s a spectrum and people don’t get that, people don’t understand that”.

(CHA01)

“There’s a lack of awareness to the wide range of visual impairment—people either think you’re blind or you’re not”.

(VIP02)

Despite this, there are strides being made to promote public awareness, especially by sight loss charities. 

#### 3.1.7. Public Transport

Stakeholders felt public transport has improved over the past few years, with staff being very helpful and understanding of people with a visual impairment.

“Improved since the improvement in buses and also the Glider—I think Translink is doing a good job at upgrading and improving”.

(PLA01)

“They say the drivers are very helpful when they are trying to get onto the bus and so on—I think from that point of its good—workers seem aware of how to deal with someone with a visual impairment”.

(OPH06)

Despite this improvement, stakeholders felt there was still a “legacy of public buses and problems with older stops” (PLA03). In addition, stakeholders agreed there is not enough rural public transport.

“No there isn’t enough public transport because I would love to be able to get to places like the Belfast Hills, certain places like that where you can walk your dog. I love walking in really gorgeous places and its really quite difficult to get there. Unless you drive a car. I would love to be able to go off to Donegal when it’s not such a mission”.

(VIP01)

“No, earlier in my career I had a PhD student who did her thesis on remote rural Ireland and built-up areas and transport—one of the big problems for people who are visually impaired and live outside the cities was transport—transport is very limited, hard to get and very few options for people to try, transport is a big issue”.

(OPH03)

Locally, in Belfast, users felt it was difficult to get from one side of town to the other

“If I wanted to go to the other side of Belfast, it’s quite hard to unless I’m getting a taxi”.

(VIP03)

### 3.2. Theme 2: The Impact of Living with Visual Impairment

Confidence impacts people’s independence, which impacts daily life and going out, which causes isolation and loneliness. This, in turn, can cause problems with people’s mental and physical health and wellbeing. 

#### 3.2.1. Confidence 

Stakeholders felt people with a visual impairment were often “reluctant to go out of their houses” (OPH06), especially older people, as they are particularly worried about trips and falls.

“Amongst older patients there’s significant anxiety around falls and trips if they can’t see things when they are on their own and out and about”.

(OPH03)

Stakeholders commented people could be “knocked off’ going out by just one bad experience.

“It doesn’t take much to knock them off”.

(OPH02)

“If you trip and fall on a pavement that hasn’t been fixed or you hurt yourself walking into a chair because somebody has put it out on a pavement—that has a big impact on your confidence and then you have to pick yourself up again and try to go out again”.

(CHA01)

There is also an embarrassment factor.

“So, if you go out and they fall then there’s an embarrassment factor saying, ‘I look stupid’ and that affects people’s confidence you know going out”.

(CHA01)

People with sight loss can also feel “vulnerable” (CHA03) and be worried about public abuse.

“People’s experience of feeling vulnerable or being shouted at or concerns about being hit by a vehicle or a cyclist”.

(CHA03)

#### 3.2.2. Or Being a Victim of a Crime

“What if someone comes up behind me and steal my purse because they know I have a sight condition”.

(CHA01)

So, they feel they are only safe using private transport, to reduce vulnerability and promote confidence.

“[There are] others who will say they will only either travel in private transport or you know taxis, not because they want to, given cost and everything but because they feel they have to”.

(CHA03)

#### 3.2.3. Independence 

Confidence issues directly impact people’s independence. If someone falls they are much less likely to go out again because they are embarrassed or have lost confidence in themselves.

“[the elderly], they are very independent, they don’t like asking for help”.

People who live rurally also find it more difficult to be independent.

“I think particularly in a rural setting that’s difficult—I think it would limit their ability to leave their house safely I would say”.

(OPH01)

In addition, shopping sites, recruitment sites, and transport sites are often inaccessible, making it even harder for people to be independent.

“How do they order their food with a website from Tesco that isn’t accessible, how do they get a job when all the recruitment websites are inaccessible, how do they work out how to get a bus when the Dublin bus website is inaccessible and you go on and on and on. That’s digital access but it permeates into the real world when pavements are accessible and you can’t find timetables to find when buses are and you have to notify Irish rail 48 hours in advance when you want some support when you’re using the train”.

(CHA02)

#### 3.2.4. Impact on Daily Life 

This confidence and potential lack of independence naturally impacts daily life, especially people’s social lives.

“It must restrict them going out”.

(PLA02)

“It must have a big impact on their lives socially”.

(PLA01)

Loss of confidence and independence makes people stay in more.

“Then people are much more likely to say I’ll not go out today because…I don’t really need that or I’ll not go to that group meeting that helps me because I know I have to manoeuvre myself round that café and I can’t really remember when it comes up”.

(CHA01)

Many feel that just leaving the house is their biggest daily anxiety or worry.

“A lot of people would say that that was their biggest daily anxiety and something they had to deal with everyday—leaving the house”.

(CHA04)

“That’s what I have to go through just to get out of the house and it’s quite hair-raising on a daily basis”.

(VIP01)

It does not have to be one big thing, it can be multiple little things such as obstacles on pavements, someone shouting something at them, or an inaccessible app.

“It’s the little barriers that you put up and then people go well I’m not going out to do that”.

(CHA01)

#### 3.2.5. Isolation (Not Going Out) and Loneliness 

Confidence, independence, and the impact on daily life often lead to isolation and loneliness. This “lack of interaction, make[s] [people] homebound” (PLA02). People start to “chose” to “sit in a lot more as time goes on and then that accumulates” (CHA01), which causes “social isolation, loneliness, and being stuck in your house” (CHA02). This can be attributed to barriers in towns and cities.

“People who say they feel trapped in their homes because public space and public realm is just not fit for purpose for them”.

(CHA03)

This is particularly prominent for “older patients” (OPH06). 

#### 3.2.6. Mental Health

Naturally, isolation and loneliness can impact people’s mental health.

“People suffer huge amounts of loneliness and isolation, mental health issues because they are literally captured prisoners in their own homes”.

(CHA02)

One interviewee quoted the following statistics.

“People with sight loss are 7× more likely to suffer from depression, a lot of that is due to social isolation, loneliness and being stuck in your house”.

Stakeholders, including visually impaired people, stated they have “anxiety” when they have to go out. The barriers of moving around the built environment do

“restrict them going out psychologically which leads to a lack of interaction, makes them homebound which impacts their physical and mental health and wellbeing”.

(PLA02)

#### 3.2.7. Physical Health 

Built environment, charities, and ophthalmic professionals talked about the impact of staying in and being isolated on physical health. Visually impaired people, however, talked about potential physical injury when encountering obstacles in the built environment.

“Bollards… I really badly whacked my knee on one where I was quite injured for a few days. They can cause serious injury and serious pain”.

(VIP03)

“I actually risk getting quite hurt when walking around so I have to be super super super careful”.

(VIP01)

#### 3.2.8. COVID-19

A majority of stakeholders agree COVID-19 has impacted people with a visual impairment. Visually impaired people said there were positives to the pandemic.

“Less mobility issues because mobility has decreased”.

(VIP03)

“There were so few cars on the road, it was the best thing to have so few cars. I felt so much safer”.

(VIP01)

COVID-19 restrictions did cause issues.

“You are trying to avoid people when you are out and about—you’re trying not to invade peoples personal space and it can be hard to see that if you’re in the aisle of a shop—it can be difficult”.

(VIP03)

“Also, with people wearing masks, it’s just generally harder to see and hear them—the overall effect of communication harder, not just for people with hearing impairment but also for people with visual impairment”.

(VIP03)

There was a level of anxiety associated with going back to normality, especially after being in isolation for so long.

“The anxiety that comes with opening up again, as someone with a visual impairment, speaking for myself, there’s part of you that likes the safety of life retreating to these bubbles, there is anxiety of going back to normal. You know when you were back to normal, you were used to going out every day and used to the mobility issues, now you have to get used to doing it all over again”.

(VIP03)

“I think it’s impacted them hugely—especially in terms of going back out again—issues surrounding them losing so much confidence with having been inside—and worries about being able to navigate places they previously code with new structures etc. confidently”.

(CHA04)

These restrictions and lockdown have also created a level of social isolation and loneliness with clinicians stating the following.

“Some of the patients we see in the clinic—that’s the first time they’ve been out of their house in months”.

(OPH01)

### 3.3. Theme 3: Policy, Regulation and Guidance 

#### Current Policy and Regulations

In general, architects and planners were aware of the policies, regulations, and guidance in place with regards to visual impairment and the built environment. Architects talked about the British Standards and code R [23], about how there are minimal requirements and visual contrast is covered “extensively” (ARC01).

Planners discussed technical documents that contain guidance “mostly for physical impairment as opposed to disabilities, such as visual impairment” (PLA01) and the Strategic Planning Policy Statement (SPPS), which “has the core planning principles and urban planning principles” (PLA03). Living places also have some guidance as an “urban stewardship design guide” (PLA03).

### 3.4. Theme 4: Future Solutions and Innovations

#### 3.4.1. The Need for Improvement

A majority of stakeholders felt there was a need for improvement in the design of our towns and cities. Many stated other countries such as Norway and Singapore are “way ahead of us” (VIP01) (ARC01).

Small things such as leading doors, contrasting steps, and considering the needs of people with a visual impairment early on could make a big difference. Architects and planners suggest there is “a lot of advice but not enough about giving actual practical examples” (ARC01).

#### 3.4.2. Potential Environmental Solutions

The overall consensus was that while there are barriers and obstacles in the built environment, these “can easily be overcome” (ARC01).

Perhaps the use of “visual contrast [and] … element contrast” (PLA01) could be implemented to make streets easier to navigate. Other potential environmental solutions include “contrast of street furniture etc. against the pavement” and “rumble strips …at traffic crossings for warning” (PLA01).

“More cycleways would mean that the three main modes of transport are separated = less people on the footway and so less danger’ (PLA02). This in turn may ease ‘congestion”.

(PLA03)

Implementation of “less street furniture, improved kerbs heights” and “[a] straight line to allow a visually impaired person to follow safely” (PLA03) could now be done in the design of the public realm and streets. In addition, increasing the width of footpaths and creating designated “furniture lines”, where furniture should be placed to allow for a safe area of the pavement, with no obstacles in the way.

#### 3.4.3. Improving Education and Awareness

Only one built environment professional did not want any more education as they felt there were already ‘endless seminars on all of this’ (ARC04).

Most built environment professionals felt that continuous professional development (CPD) modules “could be a good way of introducing education on inclusive design especially for disabilities such as visual impairment” (PLA01). In addition, professionals thought the Royal Town Planning Institute (RTPI) and the Architects Registration Board (ARB) should “require some sort of level of knowledge/education on the subject (they should set some modules you must do per year)” (PLA01).

Some built environment professionals had some practical experience in university with visual impairment, which they found beneficial.

“We had to partake in experiments within the city—Hull, England—they made us go round and see what it was like for someone with a visual impairment or wheelchair disabled”.

(ARC02)

#### 3.4.4. Professional Accountability

Stakeholders felt professional accountability was the responsibility of the architect, you have to “keep abreast of it all by reading articles and stuff like that” (ARC02). Others felt that “any improvements and problems should be identified in an auditable way to be able to truly establish it” (ARC03). Stakeholders thought there could be space for guidance documents specifically relating to visual impairment. Others felt that “in terms of the built environment we need planners to be able to take account of the wide range of lived experiences and also to understand the big issues that face many people” (CHA03).

#### 3.4.5. Open Conversations between Professionals

Stakeholders felt there needed to be open conversations and open opportunities for discussion, not only between planners and architects (ARC02), but between all stakeholders. There is a need to “work together” (CHA01).

“[there] needs to be a more open conversation between the different bodies and stakeholders that would see this as an important factor—organisations in terms of the disabled—they should become more involved with RIBA, RSUA, different architectural associations and local architects”.

(ARC02)

While these results and quotes show the multifaceted nature of navigating a built environment with a visual impairment, it is important to show the similarities and contrasts in stakeholder opinions.

All stakeholders were aware of some of the environmental barriers faced by people with a visual impairment, with all agreeing that footways have multiple issues. When cars were discussed, town planners and architects focused on issues such as transport hierarchies, while charities and visually impaired people talked about the issue of cars, speed, pedestrian safety at crossings, and the narrowing of pavements. Interestingly, electric cars were extensively mentioned by planners in a positive view, which was in contrast with the opinions of charities and visually impaired people.

Cars parked on pavements were mentioned multiple times by people with a visual impairment, charities, and ophthalmic professionals; however, they were not mentioned by planners or architects.

Colour contrast was discussed by all professionals as being important in the built environment; despite this, only one visually impaired user mentioned this briefly during their interview. Visually impaired people focused more on the issue of lighting, stating there was simply not enough and that new schemes with LED lights made it more difficult to move around as there were now gaps of lighting in between light poles. This is in contrast with planners, who thought lighting was plentiful in many areas—in fact some were creating light pollution. They were also of the opinion that these new LED lights were of benefit.

When stakeholders discussed awareness, the responses were similar. Stakeholders agreed that there were strides being taken, mainly by charities, to promote awareness of visual impairment. Despite this, there is simply not enough awareness around the spectrum and pattern of visual impairment and not “looking blind”. Everyone, except one architect, agreed awareness was lacking.

All planners and architects, except one, would have liked to see more education specifically regarding visual impairment. They would have liked to be exposed to awareness of visual impairment at the start of their professional life (in university) throughout their careers. The biggest suggestion was through CPD for architects and annual teaching for planners, preferably practical. Visually impaired individuals, charities, and ophthalmic professionals were all happy to help deliver this potential solution.

While guidance and regulation were only discussed by planners, architects, and charities, there was a call for informed, enforced, and stricter regulation and guidance to keep professionals accountable. They agreed that this needs to be achieved through conversation and agreement between all stakeholder parties.

Looking forward, all stakeholders were in agreement that there was a need for improvement, potentially by widening footpaths and reducing street furniture. Built environment professionals and charities agreed there needs to be more open conversations between stakeholders in order to improve navigation in our towns and cities for people with a visual impairment.

## 4. Discussion

The literature often describes the built environment as being “hostile” [2] and “not fit for purpose” [2]. Some common issues discussed in the interviews reflect existing literature, such as street clutter, bollards, pavement parking, and shared space areas [24,25,26]. Interviews show that visually impaired people agreed that going out into our towns and cities is “hair-raising” [9].

Another big issue was the car-centric towns and cities in which we live. Recent figures suggest Belfast is now the fifth worst congested city in the UK, with journeys taking on average 26% longer [27]. The more cars there are, the more road infrastructure we need and the less footway space there is. Footways can be very narrow, and with street furniture and other potential hazards, they can be “perilous”. In the future, NI could implement the sustainable transport approach from the National Transport Strategy, Scotland, where private cars are not a priority [28], perhaps leaving more space for footways and the people who use them.

Colour contrast could potentially improve streets for people with a visual impairment. There is little evidence on how colour contrast can improve streets for people with a visual impairment; however, interviews with the visually impaired community show enhancing colour contrast would be helpful. Suggestions include making pavements contrasting with roads or contrasting kerbs to allow for better delineation between roads and footpaths.

The benefits and disadvantages of shared space are extensively discussed in the literature [12,19,29,30]. From the interviews, it was clear built environment professionals felt the concept of shared space was not the issue, but rather people’s behaviour. The literature discusses how shared space aspires to create efficient traffic circulation, with an emphasis on the pedestrians and cyclists and an improvement in the public realm [31]. However, this is still a very contentious topic, with claims substantiated by descriptive accounts of previous schemes as opposed to academic literature [31]. It is evident from the interviews that charities do not agree that shared space is good for people with a visual impairment.

How much professionals and the general public were aware of visual impairment was discussed extensively. It was clear stakeholders did not feel there was a true awareness of the spectrum of visual impairment. Different eye problems present themselves in different ways and at different severity levels, making it very difficult for anyone to truly understand what each individual can or cannot see. This often means the general public perceive that “you are blind or you are not”, which can lead to familial problems and sometimes public harassment [32].

As expected, the potential barriers and enablers have an impact on people with a visual impairment. The interviews echoed some concerns already present in the literature. Many built environment professionals were not aware of the specifics of how visual impairment affects someone but knew it would impact their quality of life. In fact, the existing literature suggests that visual loss has the biggest impact on quality of life and is an established risk of loss of independence [13]. Discussions around people not going out of their houses or delaying going out of their houses echoed the literature on social isolation [13]. Stakeholders discussed feelings of fear and anxiety to go even the shortest of distances, with Kitchin et al. stating these feelings of fear and loss of confidence reduce exploration and independent travel in our towns and cities.

Other disabilities and co-morbidities can conflict with the needs of people with a visual impairment, which can be difficult to manage; after all, the streetscape is meant for everyone. While balancing people’s needs and wants can be difficult, consultation at the forefront of the design process can mitigate major issues. By including stakeholders in the design process [21,33], a more accessible streetscape suitable for everyone can be created. There have been challenging cases where pre-consultation was not completed or not considered and the streetscape was thus inaccessible by some users [34].

While there are legacy issues with public transport in NI, it has been improved in more recent years, with examples such as the Glider (a bus rapid transit system) [35] being implemented. Legacy issues are often caused by the Troubles with buses not being able to travel north to south or east to west in the city. The glider has improved this and now travels from outside the west of the city, to the east [36]. There have also been problems with a lack of funding for Translink, who are the public transport providers in Northern Ireland [37].

While guide dogs and other charities try to raise awareness around sight loss, with RNIB previously conducting campaigns with Channel 4 [38], in general, stakeholders agreed they wanted improved education and awareness especially on the spectrum of visual impairment. Stakeholders felt CPD for architects and a mandatory seminar for planners could be the best way to facilitate this. While many wanted this, some stakeholders claimed there were already endless seminars, and it was up to the individual to attend these. Practical seminars and teaching could allow for better understanding. In addition, facilitating professionals talking with people with varying levels and patterns of visual impairment and walking with them around a set area could be insightful.

In the future, there needs to be robust guidance and policy for planning professionals and architects. This is especially important given the COVID-19 pandemic, as pavement cafés and alfresco dining become more popular. Local councils encouraged businesses to apply for pavement cafes and alfresco dining to facilitate a reopening of urban centres [39]. These outdoor dining areas often mean more street clutter, which is problematic for people with a visual impairment. Therefore, many charities and companies have called for government action on safeguarding people with a visual impairment.

Stakeholders suggest there is a place for guidance and policy specifically related to visual impairment, including good practice examples. In addition, stakeholders suggest audits of any problems or successes could compliment the implementation of a guidance or policy document. This will also keep built environment professionals accountable while providing local good practice examples.

In addition, there needs to be open conversations between professionals and all stakeholders. With many stakeholders in charge of different areas and sections of our cities and even a single streetscape, it is vital to communicate openly. Built environment professionals need to speak with each other and not through third parties such as charities and other stakeholders in order to provide consistency in the built landscape. Built environment professionals need to consult all stakeholders at the forefront of a design proposal, leading to a people-centric design approach. Visually impaired people want to know they are thought of in the first place, and by including them from the beginning this can be achieved.

This could also be achieved through good practice community street audits, which allow for community input while allowing planners and architects to decipher community priorities and needs. The Inclusive Mobility and Transport Advisory Committee (IMTAC) offer advice and can arrange street audits while also providing “guidelines for effective consultation with older people and disabled people” [40]. These street audits and advisory boards should be contacted at the beginning of a planning consultation to make areas accessible for all.

While not discussed explicitly in these interviews, technological solutions are continuing to be developed globally, such as an electronic cane and smart glasses. In the UK and Ireland, there are no standard technological solutions for people with a visual impairment and many tend to carry a large cost. Looking into the future, technological wayfinding technology such as “smart cities” could be implemented. Guide dogs and Microsoft have previously piloted an augmented reality device called “cities unlocked”, which provides this support to visually impaired users; however, it is still in its pilot stages after several years [9].

## 5. Conclusions

In conclusion, the interviews showed that all stakeholders felt there were barriers and enablers in streetscapes for the navigation of people with a visual impairment. These barriers and enablers can profoundly impact the daily lives of people with a visual impairment, their independence, and their mental and physical health.

Despite these potential challenges, stakeholders felt small changes such as education, robust policy, and guidance and better awareness could have a big impact in making our towns and cities accessible and aesthetically pleasing to all.

While this study provided an overview of stakeholder opinions, further research into comparing issues in different cities should be completed. Street audits of different areas in different cities could be taken across a year to see how seasons, weather, etc., affect navigation. In addition, taking a practical look at certain cities over a period of time and how these issues change and fluctuate could be beneficial.

Looking towards the future, studies such as this can bring awareness to these issues, allowing further discussion between stakeholders, policy makers, and ultimately governmental bodies, who can help make a difference in improving our landscapes for people with a visual impairment. While this study was undertaken on the island of Ireland, the issues discussed are found globally; therefore, research in other countries is essential.

## Data Availability

Data can be obtained by contacting the corresponding author.
